# Consensus genetic linkage map construction and QTL mapping for plant height-related traits in linseed flax (*Linum usitatissimum* L.)

**DOI:** 10.1186/s12870-018-1366-6

**Published:** 2018-08-07

**Authors:** Jianping Zhang, Yan Long, Liming Wang, Zhao Dang, Tianbao Zhang, Xiaxia Song, Zhanhai Dang, Xinwu Pei

**Affiliations:** 1 Crop Institute, Gansu Academy of Agricultural Sciences, Lanzhou, 730070 China; 2 Institute of Biotechnology, Chinese Academy of Agricultural Sciences, Beijing, 100081 China

**Keywords:** Flax, SNP, Linkage map, QTL, Plant height, Technical length

## Abstract

**Background:**

Flax is an important field crop that can be used for either oilseed or fiber production. Plant height and technical length are important characters for flax. For linseed flax, plants usually have a short technical length and plant height than those for fiber flax. As an important agronomical character for fiber and linseed flax, plant height is usually a selection target for breeding. However, because of limited technologies and methods available, there has been little research focused on discovering the molecular mechanism controlling plant height.

**Results:**

In this study, two related recombinant inbred line (RIL) populations developed from crosses of linseed and fiber parents were developed and phenotyped for plant height and technical length in four environments. A consensus linkage map based on two RIL populations was constructed using SNP markers generated by genotyping by sequencing (GBS) technology. A total of 4497 single nucleotide polymorphism (SNP) markers were included on 15 linkage groups with an average marker density of one marker every 2.71 cM. Quantitative trait locus (QTL) mapping analysis was performed for plant height and technical length using the two populations. A total of 19 QTLs were identified for plant height and technical length. For the MH population, eight plant height QTLs and seven technical length QTLs were identified, five of which were common QTLs for both traits. For the PH population, six plant height and three technical length QTLs were identified. By comparing the QTLs and candidate gene information in the two population, two common QTLs and three candidate genes were discovered.

**Conclusions:**

This study provides a foundation for map-based cloning of QTLs and marker-assisted selection for plant height-related traits in linseed and fiber flax.

**Electronic supplementary material:**

The online version of this article (10.1186/s12870-018-1366-6) contains supplementary material, which is available to authorized users.

## Background

Flax (*Linum usitatissimum* L.) is an annual self-pollinated diploid crop (2n = 2× = 30) that can be used either for stem fiber or seed oil production about 7000 years ago [1]. Fiber flax and linseed flax are genetically the same but morphologically different [2]. One of the important differences between fiber and linseed flax is the character of plant height. Fiber flax is usually 80–120 cm high with fewer branches, while oil flax is usually about 70 cm high with many branches. Fiber flax is mainly grown in Northern Europe, Russia and China, and linseed flax is widely grown in Canada, India, America, Argentina and Germany, as well as Russia and China (Scientific Database of China Plant Species, http://db.kib.ac.cn). Flax oil contains a mixture of fatty acids, including saturated, monounsaturated and polyunsaturated fatty acids. Among the different types of fatty acids, the oil is rich in polyunsaturated fatty acids, particularly alpha-linolenic acid (ALA), the essential omega-3 fatty acid, and linoleic acid (LA), the essential omega-6 fatty acid [3]. In China, linseed flax is mainly distributed in the Northwest and North areas, and has a planting history of about 2000 years [4].

As one of the important agronomical characters for both fiber and linseed flax, plant height is usually a selection target in breeding. Plant height trait has been found to have a significant co-relationship with seed yield traits, such as seed weight. For example, Contreras-Soto et al. found that some SNP markers on Chr19 controlled both the plant height and seed weight traits through a genome-wide association study (GWAS) in soybean [5]. Technical length is another selective breeding target in flax. In flax, Soto-Cerda et al. used 464 simple sequence repeat (SSR) markers to genotype 390 accessions and investigated the phenotypes of nine agronomic traits. Through GWAS analysis they discovered that 12 markers were significantly associated with six traits [6]. Halbauer et al.,(2017) used gSSRs to analyze 27 flax (*Linum usitatissimum* L.) accessions originating in the Alpine region and found a varying extent of accession-specific gene diversity (expected heterozygosity, HE) was revealed ranging from 0.05 to 0.51 [7]. However, there has been no research on plant height and technical length gene discovery in flax to date.

In conjunction with genomic tool development, a linkage map and QTL mapping are useful tools for discovering genes controlling important agronomic traits [8–10]. In flax, only a few linkage maps have been published. The earliest linkage map was constructed with 213 RAPD and RFLP markers in 18 linkage groups, and two Fusarium wilt QTLs were identified [11]. Later, Oh et al. also used RAPD and RFLP markers to construct a linkage map composed of 94 markers [12]. With the development of molecular markers, SSRs have become the major marker type for linkage map construction. Cloutier et al. constructed a linkage map that included 24 linkage groups with 113 EST-SSR markers for a DH population, and identified QTLs for seed color, linolenic acid content and linoleic acid content [13]. Then several maps were constructed based on SSR markers. For example, Cloutier et al. construct a consensus linkage map by combined three individual linkage maps incorporating 770 markers based on 371 shared markers including 114 that were shared by all three populations and 257 shared between any two populations [14]. Most of the QTL mapping analysis focused on fatty acid related traits, such as fatty acid composition, yield [15, 16], seed and flower color [17]. For example, Sudarshan et al.,(2017) identified a seed and flower color QTL “D” by QTL mapping analysis and identified a candidate gene [17].Today, SNP markers are the most efficient and abundant markers for mapping [18] and other applications, such as GWASs [19, 20], diversity analyses [21] and bulked segregate analysis [22].

Most SNP markers come from sequencing data generated by high-throughput sequencing technologies using genomic sequences as references. Whole genome sequences have been determined for a range of species, including soybean, *Brassica rapa*, cotton, flax and about other 60 plants (https://phytozome.jgi.doe.gov/pz/portal.html). The flax genome includes about 302 Mb of non-redundant sequence representing an estimated 81% genome coverage [23]. However, the sequence data have only been assembled into 88,384 scaffolds containing 43,384 genes, and these are not well anchored to a linkage map showing the relative positions of the genes. Recently, many new technologies have been developed for SNP marker discovery, such as genotyping-by-sequencing (GBS) [24, 25], restriction site-associated DNA (RAD) sequencing [26, 27], SLAF-seq (specific length amplified fragment sequencing) [28] and ddRAD sequencing [29]. In flax, GBS [30] and SLAF-seq [31] technologies have been used for SNP marker identification. Kumar et al.,(2012) constructed eight reduced representation libraries to do GBS and discovered 55,465 SNPs in the genome of flax [27] and then used 329 SNP and 362 SSR markers to construct a linkage map and identified a total of 20 QTLs corresponding to 14 traits. Yi et al.,(2017) used a F2 population to do SLAF-seq and construct a genetic map for flax and finally they discovered 4638 SNPs for genetic map construction. The final genetic map included 4145 SNP markers on 15 linkage groups and with 2632.94 cM in length [31].

Thus, linkage map construction based on SNP markers has become easier and more efficient. Accordingly, candidate gene analysis for important agronomic traits has also become easier. For example, Singh et al. identified a candidate gene for 100-seed weight and root/total plant dry weight ratio under rainfed conditions in chickpea using whole genome resequencing and variant SNP loci collection [32]. Using recent tools for SNP marker identification, the aims of this study were to: 1) develop SNP linkage maps for two related recombinant inbred line (RIL) populations; 2) construct a consensus linkage map based on the two linkage maps; 3) perform QTL mapping for the plant height and technical length traits in the two related populations.

## Results

### Phenotype variation in the two populations

**Fig. 1:**
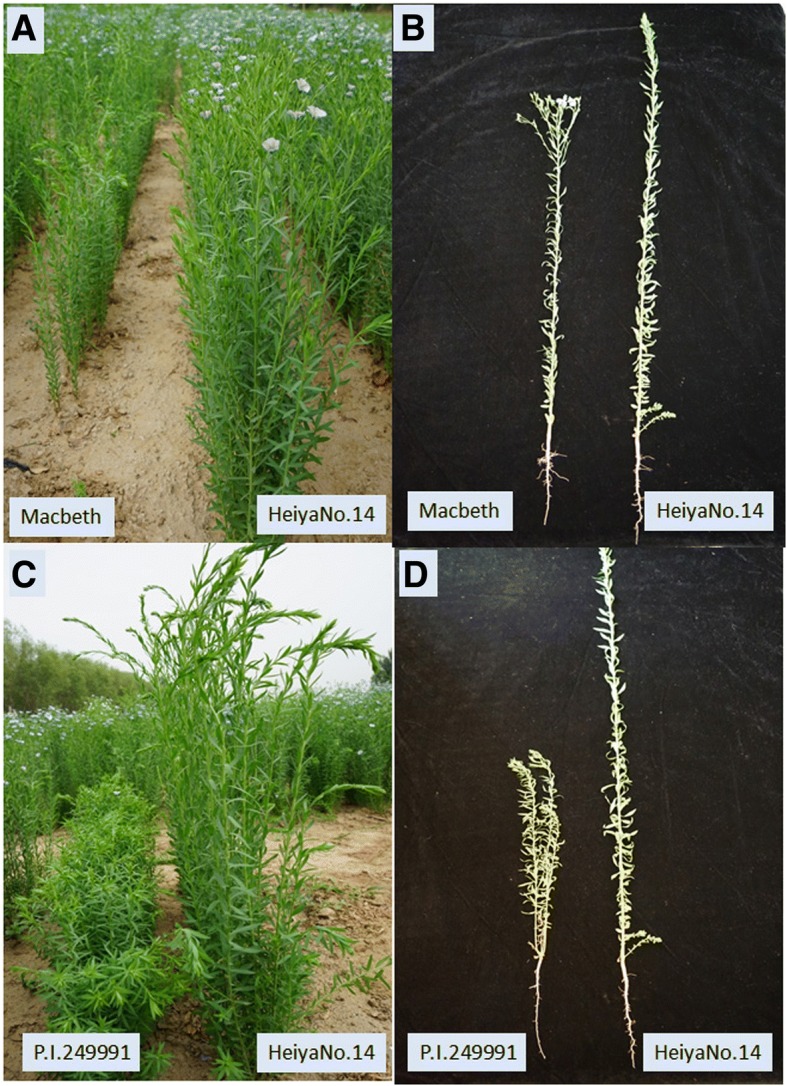
Plant height phenotypes for the parents in the two populations. **a**, **b** Plant height phenotypes for the two parents, Macbeth and Heiya No.14, in the MH population; **c**, **d** plant height phenotypes for the two parents, P.I.294441 and Heiya No.14, in the PH population

**Fig. 2:**
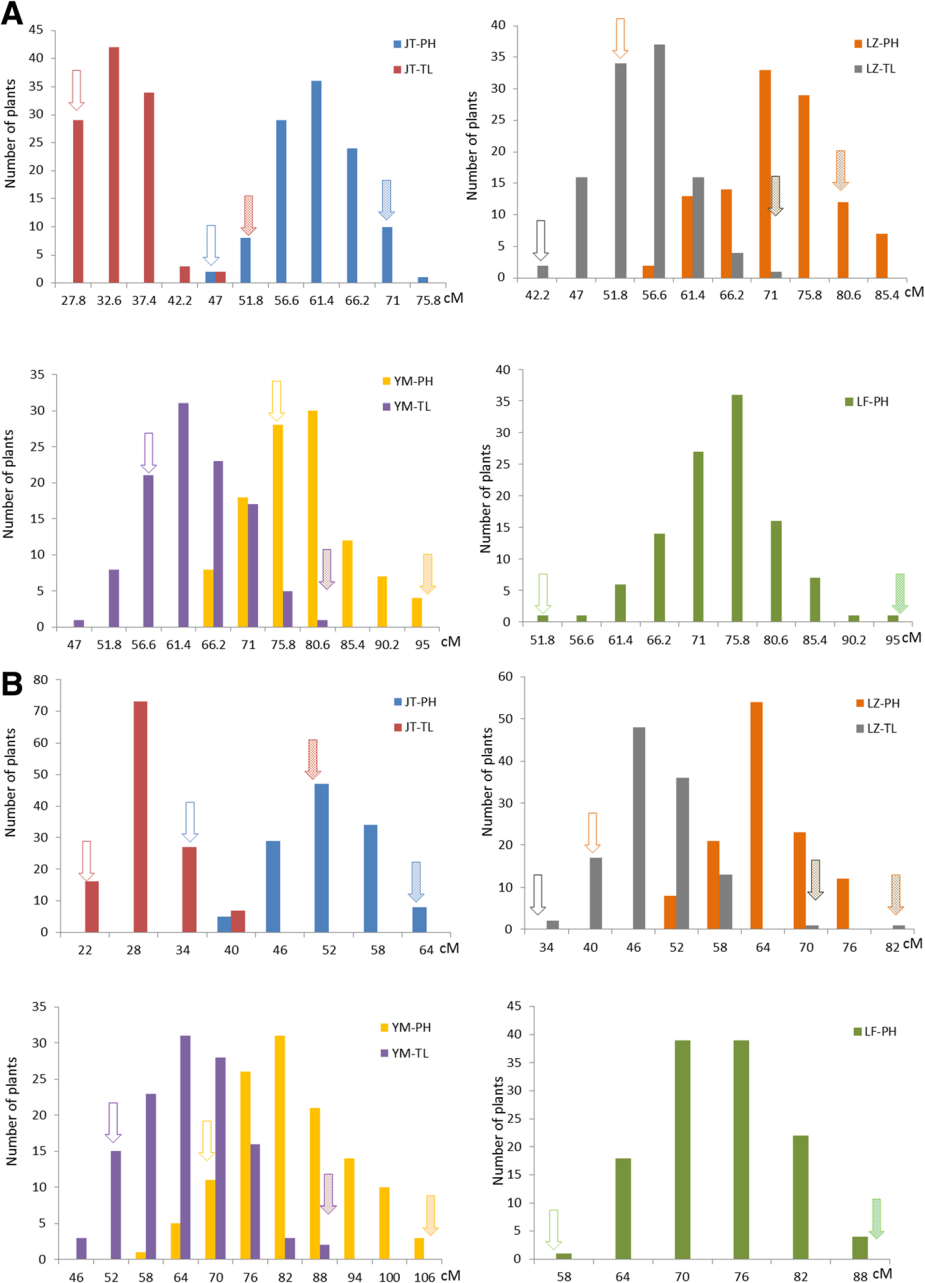
Plant height and technical length distributions in different environments in the two populations. **a** PH distribution in the MH population. Solid arrows represent the female parent Macbeth, and arrows with oblique lines represent the male parent Heiya No.14. **b** PH distribution in the PH population Solid arrows represent the female parent Macbeth; arrows with oblique lines represent the male parent Heiya No.14. Solid arrows represent the female parent PI.29991, and arrows with oblique lines represent the male parent Heiya No.14

For the plant height and technical length phenotypes, the common male parent Heiya No.14 of the two populations showed higher values than both of the female parents, Macbeth and P.I.249991 (Fig. 1). The plant height and technical length values of the individuals in the two populations showed normal distributions in all of the environments (Fig. 2). The correlation analysis showed that the two traits in the two populations were positively related, and the correlation coefficient ranged from 0.7–0.85 in different environments. The phenotypic variations of the two related populations were listed in Table 1. An interesting phenomenon was that, for plant height, the phenotypic values were lowest in Jingtai, followed by Lanzhou and Langfang, and highest in Yuanmou (Table 1, Fig. 2). The same tendency was observed for the technical length distribution in three of the environments.

**Table 1:** Phenotypic variation of MH and PH population in four environments

Trait	environments	Female (cm)	Male (cm)	Min^a^ (cm)	Max ^b^ (cm)	Mean^c^ (cm)	SD^d^	CV (%)^e^	Kurto sis^f^	Skewness^g^
PH population										
PH	LZ	38.20	81.80	48.90	75.40	62.15	5.76	9.27	0.13	−0.14
	JT	29.65	63.68	35.50	62.65	49.98	5.46	10.92	0.03	−0.52
	YM	65.50	110.60	55.70	105.80	78.97	9.77	12.37	0.06	−0.06
	LF	47.67	103.56	50.78	85.00	69.87	6.51	9.32	−0.24	−0.16
TL	LZ	27.50	70.30	31.70	81.20	56.45	5.99	10.62	1.64	0.40
	JT	20.86	48.38	16.50	41.40	28.95	4.52	15.63	0.90	1.31
	YM	51.32	88.30	38.60	128.00	83.30	10.39	12.47	1.63	0.30
MH population										
PH	LZ	51.00	80.50	51.90	84.70	68.75	6.73	9.78	0.07	−0.44
	JT	49.40	70.75	43.30	82.00	58.53	5.99	10.23	0.57	0.89
	YM	74.10	110.60	59.40	94.70	75.19	7.35	9.77	0.27	−0.33
	LF	50.90	93.50	50.78	94.78	70.01	7.89	11.26	−0.11	0.02
TL	LZ	41.87	68.90	35.40	67.00	51.09	5.82	11.39	0.05	−0.36
	JT	26.30	53.50	23.15	50.75	30.65	4.43	14.45	0.84	1.98
	YM	55.10	80.00	46.40	75.90	60.04	6.70	11.16	0.07	−0.58

^a^Max(cm): The maximum value of phenotypic data in the two RIL populations

^b^Min(cm): The Minimum value of phenotypic data in the two RIL populations

^c^Mean(cm): The average value of phenotypic data in the two RIL populations

^d^SD: Standard deviation of the phenotypic trait

^e^CV (%): Coefficient of variation of the phenotypic trait

^f^Skewness: Skewness of the phenotypic trait

^g^Kurtosis: Kurtosis of the phenotypic trait

### ddRADseq statistics for the two RIL populations

**Fig. 3:**
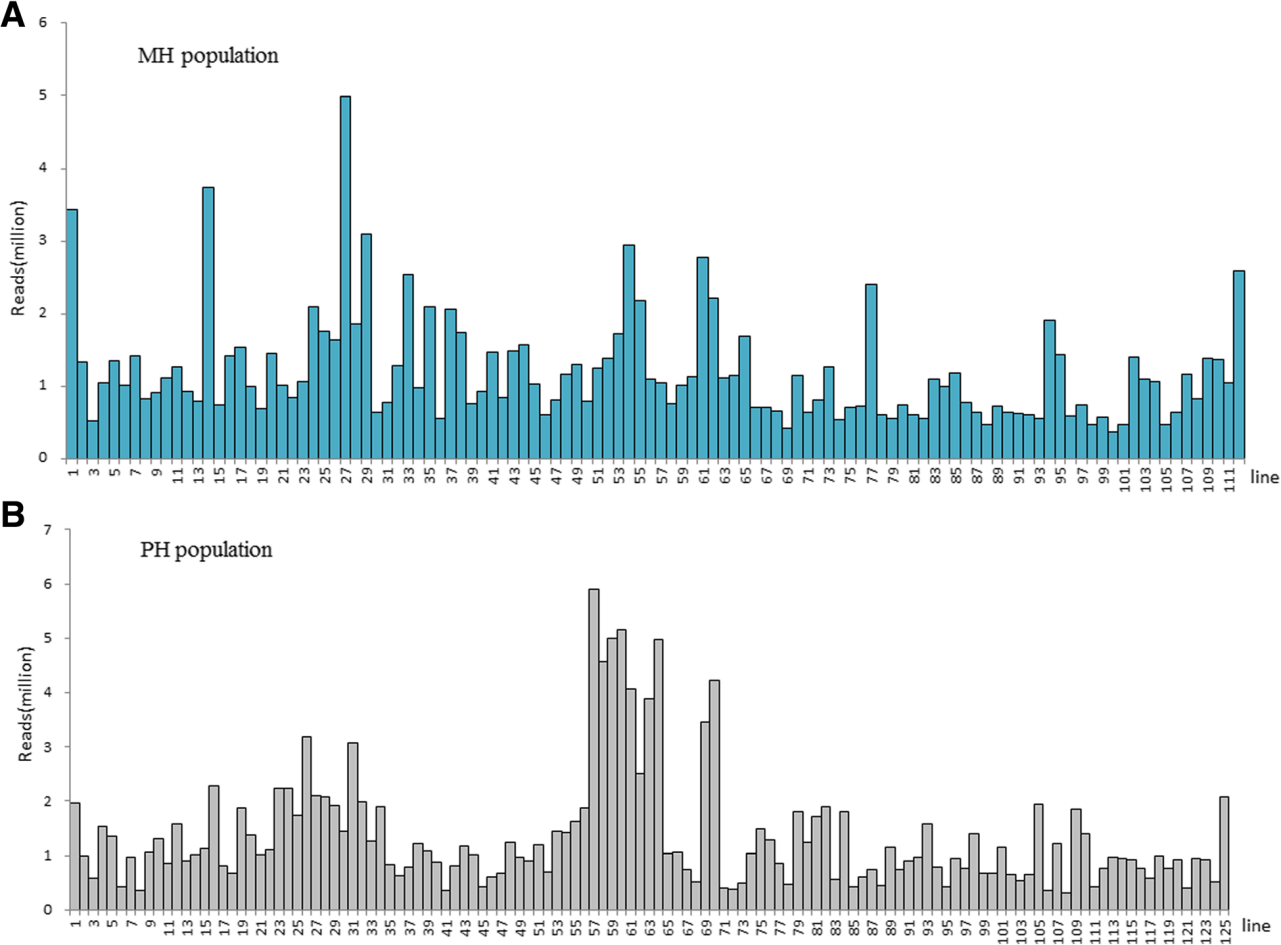
The number of sequence reads for the two populations. The characters in X-axis mean the number of lines and the Y-axis represents the reads number for each line. **a** MH population. The number 1–110 in X-axis represent the RIL number, and number 111 mean the female parent Macbeth, 112 mean the male parent Heiya No.14. **b** PH population. The number 1–123 in X-axis represent the RIL number, and number 124 mean the female parent P.I.249991, 125 mean the male parent Heiya No.14

The ddRAD seq protocol was used to construct sequence libraries for both the MH and PH RIL populations. The genomic DNA was double digested with the restriction enzymes *Sac*I and *Mse*I, and then fragments in a size range of 141–420 bp were recovered. Libraries from 12 different individuals tagged with 12 barcodes were pooled and sequenced on the Illumina HiSeq2000 platform. The three parents Macbeth, Heiya No.14 and P.I.249991 yielded 1.05(3.5× genome coverage), 2.59 (8.6× genome coverage) and 2.08 (6.9× genome coverage) million PE reads, respectively. For the MH population, the 110 individuals yielded a total of 135.95 million PE reads, ranging from 0.38(1.3× genome coverage) to 4.99 (16.6× genome coverage) million reads in different RILs with an average of 1.21(4.03× average genome coverage) million reads per RIL line (Fig. 3a). For the PH population, the 123 individuals yielded a total of 168.19 million PE reads, ranging from 0.31(1.03× genome coverage) to 5.9 (19.7× genome coverage) million reads in different RILs with an average of 1.37 (4.57× average genome coverage) million reads per RIL line (Fig. 3b).

### SNP genotyping and linkage map construction in the two RIL populations

After getting the raw sequencing data, three major steps were used to identify SNP markers. First, the 5 bp index at the end of 5′ end was removed and the last 5 bp error-enriched nucleotides at the 3′ end were trimmed for each read. Therefore, 80 bp PE reads were kept for the following analysis. Then, subsequent steps including recognizing the tags, constructing tag networks, removing erroneous tags and filtering out networks having only a single tag were done. Finally, 7399 and 5505 SNPs were identified in the PH and MH populations, respectively. The distribution of informative SNP numbers was 24.5 and 18.2 per megabase along the flax chromosome for each population. This mean that the polymorphic variation between Macbeth and Heiya No.14 was lower than the two parents in PH population.

To construct the linkage maps, one SNP was selected from each allelic tag pair to represent the locus, which resulted in a set of 4348 and 3231 SNPs for the PH and MH population, respectively. After deleting the distorted SNP markers, finally a total of 2788 SNP markers distributed on 15 linkage groups were mapped for PH population, with a length of 1138 cM. The average marker density was 2.45 markers/cM. The LGs ranged from 15 to 137 cM and contained 36 to 329 markers. For the PH map, a total of 2120 segregating markers were mapped on 15 linkage groups, and the total length was 1272 cM. There were 34 to 267 markers distributed on different LGs. The average marker density was 1.67 markers/cM. All the information for the SNP linkage maps is listed in Table 2. By combining the two individual linkage maps, an integrated linkage map was constructed. In total, the integrated map included 4497 molecular markers and was 1658 cM long. For each LG, the marker number ranged from 154 to 576 (Table 2, Additional file 1, Additional file 2). The biggest linkage group was *Lu9* with 451 integrated markers, while the smallest linkage group was 87 cM and contained 154 molecular markers. Then the flanking sequences (Additional file 2) of all of the 4497 SNP markers were used for BLAST with the published flax genome. Totally, 1996 scaffolds were identified, and among them 1493 scaffolds were non-redundant. The other 2501 SNP markers couldn’t be mapped to the reference genome, which mean that genetic differences existed in different flax cultivars.

**Table 2:** Mapping statistics for the three individual and the consensus genetic maps of flax

Linkage group	MH map			PH map			Integrated		
	marker number	Length (cM)	average distance(cM)	marker number	Length(cM)	average distance(cM)	marker number	Length(cM)	average distance(cM)
*Lu1*	88	46	0.52	175	75	0.43	257	83	0.32
*Lu2*	36	15	0.42	204	119	0.58	238	124	0.52
*Lu3*	128	63	0.49	210	105	0.50	333	108	0.33
*Lu4*	143	67	0.47	37	57	1.55	177	74	0.42
*Lu5*	187	90	0.48	95	65	0.68	267	110	0.41
*Lu6*	213	72	0.34	148	52	0.35	334	81	0.24
*Lu7*	122	59	0.49	34	50	1.47	154	67	0.43
*Lu8*	329	104	0.32	267	141	0.53	576	167	0.29
*Lu9*	258	112	0.43	199	128	0.64	451	184	0.41
*Lu10*	184	69	0.38	90	54	0.60	261	111	0.43
*Lu11*	100	57	0.57	100	97	0.97	192	102	0.53
*Lu12*	159	53	0.34	148	65	0.44	298	83	0.28
*Lu13*	348	137	0.39	214	122	0.57	523	171	0.33
*Lu14*	240	91	0.38	40	58	1.45	277	107	0.39
*Lu15*	253	103	0.41	159	84	0.53	159	84	0.53
Total	2788	1138	0.41	2120	1272	0.60	4497	1658	0.37

### QTL mapping of plant height and technical length in the two RIL populations

**Fig. 4:**
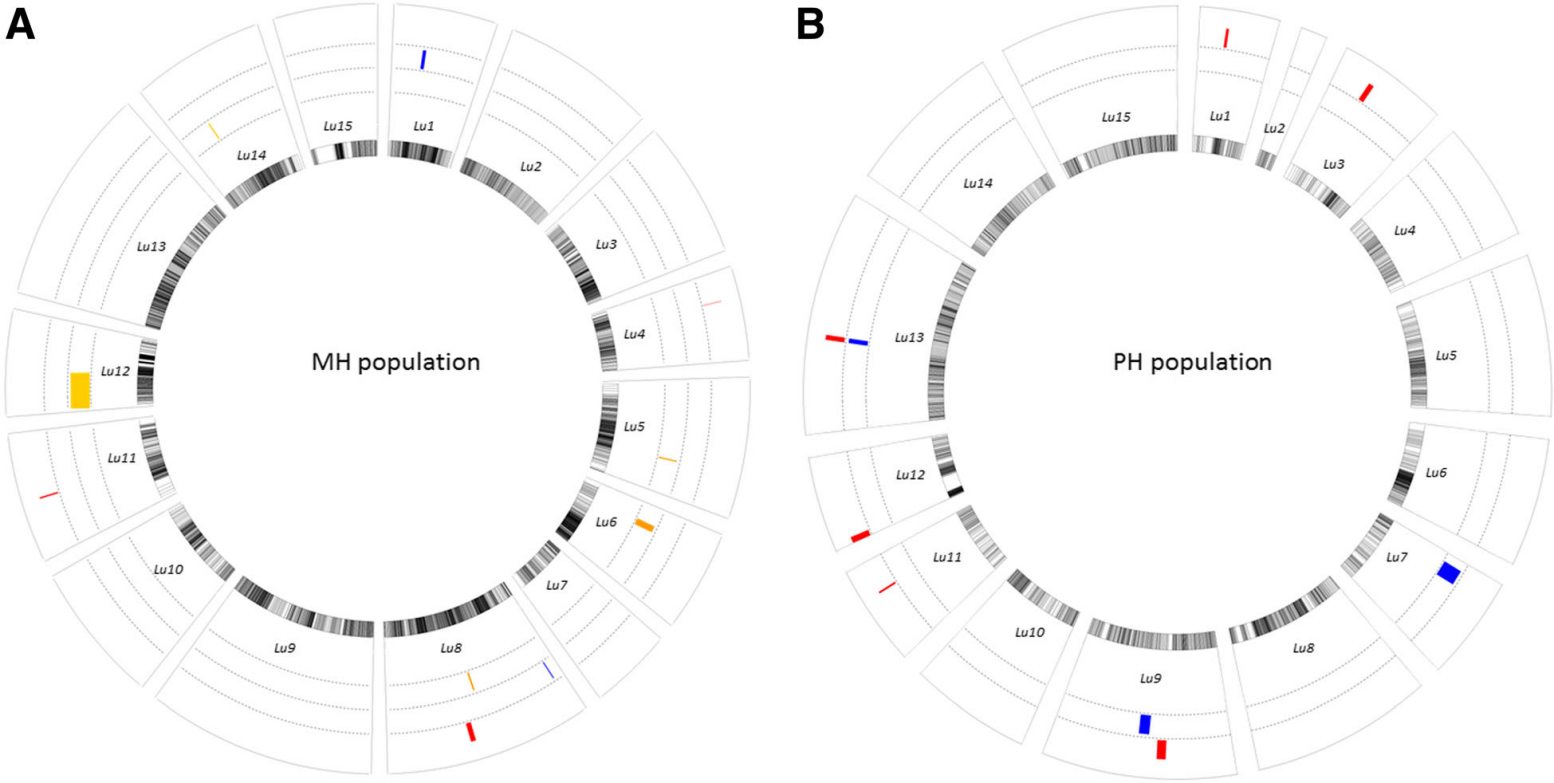
Graph of QTL mapping results for the two populations. **a** MH population. **b** PH population. The innermost circle represents 15 linkage groups, the outer circles represent QTL mapping results for plant height and technical length. Additional data about of the genetic maps can be found in Additional files 1 and 2. The circle with a red bar shows the QTLs for plant height, the circle with a blue bar shows the QTLs for technical length, and the circle with an orange bar shows QTLs detected for both of the traits

QTL mapping analysis was done using the WinQTLcart2.5 software. After initial QTL mapping analysis, several plant height and technical length QTLs were identified in different environments in the two populations (Table 3, Fig. 4). For the MH population, 10 QTLs distributed on eight linkage groups were identified, the phenotypic variation ranged from 18 to 26%, and most of the additive effect was negative, which means that most of the alleles from the female parent Macbeth made the plants shorter. For the plant height trait, eight QTLs distributed in seven linkage groups were identified. Seven QTLs on six linkage groups were detected for the technical length trait. Comparing the QTLs of the two traits showed that five QTLs were shared by both traits, such as *uq.C5–1* and *uq.C6–1*. Conversely, three unique QTLs were detected in multiple environments and the other seven QTLs were environment-specific. For the PH population, nine QTLs distributed on eight linkage groups were identified, and the phenotypic variation ranged from 18 to 68% (Table 2, Fig. 4). Six QTLs distributed in six linkage groups were identified for the plant height trait, and three QTLs on three linkage groups were detected for the technical length trait. Comparing the QTLs of the two traits showed that no QTLs controlled both traits. Additionally, only one unique QTL was detected in two environments; the other QTLs were environment-specific. When the QTL mapping results were compared between the two populations, only one QTL located in *Lu11* was detected in both populations. After the QTL mapping analysis, the candidate genes of the QTLs were identified based on the sequence information of the SNP markers. In total, 28 potential flax candidate genes were located in the QTL confidence intervals, including 17 candidate genes for the MH population and 11 candidate genes for the PH population (Table 3). Gene annotation results showed that different gene functions were potentially involving in the plant developing in flax, such as LACCASE, MYB regulators. And the actual functional genes controlling the plant height and technical length QTLs would be discovered by further study.

**Table 3:** QTL information for plant height and technical length in two populations

QTL name	LG	Position	Closet marker	LOD	Additive	R^2^	LOD2_L	LOD2_R	Trait	Environment	Candidate gene	Gene annotation
MH population												
*uq.C1–1*	1	28.51	Lu1_695389	3.71	1.57	12.76	27.5	30.5	TL	Yuanmou	*Lus10012562,Lus10016188*	Unknown function; COBRA-like protein
*uq.C4–1*	4	24.41	Lu4_300701	3.07	2.28	25.7	24.1	24.4	PH	Langfang	*Lus10041481*	Multicopper oxidase
*uq.C5–1*	5	52.51	Lu5_8504	3.89	1.95	25.31	52.2	53.3	PH,TL	Langfang, Jingtai	*Lus10007137*	large subunit ribosomal protein L13Ae
*uq.C6–1*	6	19.81	Lu6_639236	3.63	1.79–2.03	16.21–22.47	17.8	24	PH,TL	Yuanmou	*Lus10034082,Lus10017587, Lus10019435*	Proline-rich receptor-like protein kinase perk; carboxylesterase 17-related;oxidoreductase
*uq.C8–1*	8	10.11	Lu8_646184	3.62	1.25	8.16	9.6	10.4	TL	Jingtai	*Lus10011957*	nucleosome assembly protein 1-like 1
*uq.C8–2*	8	81.21	Lu8_185009	5.09	−2.16	19.08	80.6	82.1	PH,TL	Jingtai		
*uq.C8–3*	8	93.41	Lu8_119488	3.14	−1.97	25.98	92.2	96.5	PH	Langfang	*Lus10012249,Lus10012252*	fanconi anemia group J protein;high mobility group protein 2-related
*uq.C11–1*	11	46.91	Lu11_557617	3.3	−1.88	22.19	46.6	47.7	PH	Langfang	*Lus10038920*	monocopper oxidase-like protein sks1-related
*uq.C12–1*	12	34.31	Lu12_696508	2.7–4.8	−4.53	18.71–23.54	2.8	36.7	PH,TL	Langfang, Lanzhou, Yuanmou	*Lus10036228,Lus10036227,Lus10036229, Lus10036247,Lus10023392*	Ribosomal protein L7/L12-related; UDP-N-acetylglucosamine pyrophosphorylase; choline-phosphate cytidylyltransferase; F21O3.9 Protein-related;protein LSD1
*uq.C14–1*	14	32.61	Lu14_231853	3.94–7.26	2.56–3.08	20.62–26.94	32	33.1	PH, TL	Langfang,Lanzhou, Yuanmou	*Lus10015608,Lus10015614*	MYB domain protein 26; insoluble isoenzyme cwinv1-related
PH population												
*uq.C1–1*	1	17.61	Lu1_396428	3.79	−1.84	21.11	16.2	17.9	PH	Langfang	*Lus10027782,Lus10016188, Lus10027887*	Laccase-10-related;COBRA-related protein 6; Wax ester synthase-like Acyl-CoA acyltransferase domain; Wax ester synthase-like Acyl-CoA acyltransferase domain
*uq.C2–2*	2	96.71	Lu2_597057	6.79	1.77	10.29	94.6	97.9	TL	Jingtai	*Lus10014561,Lus10014535, Lus10030692*	glycosylphosphatidylinositol transamidase; Formate dehydrogenase
*uq.C3–1*	3	23.41	Lu3_693423	4.05	−2.42	20.53	20.9	24.2	PH	Lanzhou		
*uq.C7–1*	7	12.31	Lu7_781312	3.04	3.67	73.41	5.8	15.7	TL	Yuanmou		
*uq.C9–1*	9	41.41	Lu9_503128	6.43	2.68	21.83	40.9	46.4	PH	Yuanmou, Langfang	*Lus10002031,Lus10015623*	Unknown function; fatty acid omega-hydroxy dehydrogenase
*uq.C9–2*	9	56.21	Lu9_618122	3.43	1.22	10.53	51.9	58.7	TL	Jingtai		
*uq.C11–1*	11	37.41	Lu11_447048	4.54	−2.32	22.91	36.9	38	PH	Langfang	*Lus10038920*	monocopper oxidase-like protein sks1-related
*uq.C12–1*	12	2.01	Lu12_163596	3.44	−2.23	21.84	0	3.6	PH	Lanzhou	*Lus10031416*	Nicotianamine synthase
*uq.C13–1*	13	54.91	Lu13_367183	3.34	3.21	68.2	53.8	56.7	PH	Yuanmou	*Lus10026439,Lus10026441*	Threonine-specific protein kinase; peptidyl-prolyl cis-trans isomerase SDCCAG10

LOD2_L/R: Confidence interval in 95% significance level; TL: technical length; PH: Plant height

## Discussion

In the current study, a high-density integrated linkage map was constructed based on individual linkage maps from the MH and PH populations. Differing from previously published linkage maps in flax, the molecular markers used in this study were SNP markers based on the high-throughput sequencing technology-ddRAD sequencing method. Compared with traditional RAD sequencing, the ddRAD method simplifies the experimental process and reduces the cost of reduced representation library construction, and can be used in species without a reference genome [33]. Poland et al. first used this technology to develop thousands of SNP markers and constructed two high-density linkage maps for barley and wheat [34]. Subsequently, ddRAD-derived SNP linkage maps were constructed in different species, such as peanut [35] and strawberry [36]. In flax, the first SNP maps were developed by the GBS technology. Kumar et al., (2012) first used GBS technology to discovering SNPs among 8 flax cultivars [30], meanwhile Cloutier et al., (2012) constructed consensus linkage map by using these SNP markers and SSR markers, and finally there were 770 markers distributing in 15 linkage groups [14]. Until now, the latest linkage map published by Kumer et al.,(2015) was with 329 SNPs and 362SSRs. Compared with the previous linkage maps, the current SNP map generated in this study is the most high-density map to date and will help flax researchers to discover flax genes more easily.

Based on the two individual linkage maps, a consensus linkage map was constructed. Mapping of genetic markers using multiple populations provides increased genome coverage because it is unlikely that multiple parents would all be fixed (monomorphic) in the same genomic regions. In the MH linkage map, the *Lu2* linkage group contained only 36 molecular markers and was 15 cM, whereas in the PH linkage map, there were 204 molecular markers in the *Lu2* linkage group covering 119 cM. Thus, once these two linkage groups were integrated together, the coverage of this group was greatly increased. Additionally, we anchored the published scaffolds onto the consensus map based on the SNP sequencing information. In total, 1493 non-redundant scaffolds were mapped onto the linkage map, which means that these scaffolds were physically mapped onto the linkage map and will help to construct the whole physical map. While there were still 2501 SNP markers couldn’t be anchored with published reference genome sequences. One of the reasons was genetic differences existing between the reference genome and the current flax cultivar genomes. The other possible reason was that the coverages of the reference genome published in 2012 [23] were not enough to dissect the whole flax genome. So the consensus linkage map would be benefit for flax genome assembly. Additionally, the flanking sequencing information could help to discover candidate genes for target QTLs of specific traits.

Plant height is an important agronomic trait affecting crop performance, particularly lodging and consequently yield and quality. Technical length is also an important trait for flax breeding. Previous studies have shown that plant height and technical length are positively correlated. In general, flax plants with a greater technical length also have a greater plant height. There are different demands for plant height and technical length in flax because of its different usages. Specifically, fiber-type flax needs to be taller, while linseed-type flax needs to be relatively short. Thus, different alleles that can enhance or reduce plant height and technical length phenotypes are needed according to different breeding purposes for flax. In this study, the phenotype of plant height showed obvious differences among the four environments. Additionally, the same phenotype distribution was found in three locations. The average value of plant height was smallest in Jingtai, followed by Lanzhou and Langfang, and largest in Yuanmou. This was because of the latitude, longitude and temperature differences in these locations. It was easy to understand why the plant height and technical length were highest in Yuanmou. We planted the two populations in October and the growth cycle was 5 months long, which was much longer than in the other three locations. In the other three locations, we planted the populations in April and harvested the seeds in early July, so the life cycle was only 3 months. Among these three locations, plant height was greatest in Langfang, followed by Lanzhou and lastly Jingtai, which means that plant height was positively correlated with longitude and negatively correlated with latitude. Several previous studies have shown that different environmental factors can influence plant height. For example, a photoperiod insensitive allele of the major photoperiod regulator *Ppd-1*, located on group 2 chromosomes, can have pleiotropic effects on plant height [37]. In flax, Soto-Cerda et al., (2014) used GWAS approach to identify genes relating to 9 agronomic traits including plant height. The results showed that one SSR marker was not only relate with flowering time, also relate with plant height [6]. We then compared the QTLs got from the GWAS analysis and from the current study, it was found that the QTLs were not same in the two studies. The reason was maybe the different genetic background of the research materials. Besides comparing the results from two different studies, we compared the QTL mapping results from the two populations. The results showed that most of the QTLs were unique, meaning that the QTLs were significantly influenced by the genetic background. One unique QTL *uq.C11–1* located in *Lu11* was detected in both populations, and the candidate gene *Lus10038920* was identified. This showed that a common candidate gene contributed to the plant height trait in both of the populations. Thus, we could focus on this QTL and try to use the sequence information from the SNP markers for MAS in flax.

## Conclusions

Flax is an important oilseed crop over the world, while until now there were few researches about gene and genomic information. In the current study, two RIL populations were used as materials to generate SNP markers and investigate the genetic control of plant height-related traits. As a result, a consensus high density linkage map was constructed based on the two individual linkage maps. Based on the linkage map and phenotypes, QTL mapping analysis was done for plant height and technical length. Totally there were 19 QTLs were identified. For MH population, eight plant height QTL and 7 technical length QTL were identified, and 5 are common QTLs. For PH population, there were 6 plant height QTL and 3 technical length QTL respectively. After comparing the QTL and candidate gene information of the two populations, two common QTLs and three candidate genes were discovered. This study provides the foundation for assisting in map-based cloning of the QTL and marker assisted selection of plant height related traits in linseed and potentially fibre flax.

## Methods

### Plant materials

Two populations were used for linkage map construction and QTL mapping. The first was a Macbeth/Heiya No.14 (MH) RIL population. The MH RIL population RILs was developed from.

“Macbeth×Heiya No.14” by single-seed descent in seven generations. Macbeth is a Canadian ‘conventional’ oilseed-type cultivar with [38] 55–57% linolenic acid, and Heiya No.14 is a Chinese fiber cultivar [39]. In total, 110 RILs were randomly selected from the original 235 lines and were used for genotyping and phenotyping analysis.

The second population was a P.I.249991/Heiya No.14 (PH) population, which consisted of 123 R7-derived RILs. The RILs were generated from a cross between the oilseed-type P.I.249991 with about 53% linolenic acid [40] and the Chinese fiber flax variety Heiya No.14 by single-seed descent. As with the MH population, the plant height phenotypes of the two parents were significantly different (Fig. 1). The two populations were developed in Crop Institute of Gansu Academy of Agricultural Sciences.

### Field trial experiments and phenotype measurements

The two populations and their parents were grown in four different locations over two years (Additional file 3). In 2015, the two populations were planted in the fields of Crop Institute of Gansu Academy of Agricultural Sciences, Lanzhou and Jingtai in Gansu Province of China, and Yuanmou in Yunnan Province. Then, the two populations were planted in the fields of Biotechnology institute of Chinese Academy of Agricultural Sciences, Langfang in Hebei Province in 2016. Three replications were included in each of the field trial experiments. Three lines including about 30 plants were included in each plot. Each plot was with 1 m long and 0.6 m wide. Five plants were randomly selected for plant height (PH) and technical length (TL) measurements with unit cm. The plant height was measured from the bottom to the top of the whole plant, and the technical length was referred to the length of the main stem, that mean the length from the bottom until the first branch. PH values were measured in all four environments, and TL values were recorded in three environments. Then the average values, standard deviation, coefficient of variation, skewness and kurtosis of each trait were analyzed by SPSS17 software.

### DNA extraction, library construction and sequencing

Genomic DNA was extracted from young leaves of a single plant for each inbred line of each RIL population. The extraction method was as described by Murray and Thompson [41]. The DNA quality was checked with a Nanodrop2000 and by running it on a 1% agarose gel. Then, 200 ng qualified genomic DNA was used for sequence library construction and SNP marker development.

The double digested restriction site-associated DNA (ddRAD) sequencing method was used for sequence library construction and SNP marker development. ddRAD libraries for the parents and all RILs were constructed as described previously [29]. First, the 200 ng DNA was digested with two restriction endonucleases *SacI* and *MseI*, and then adaptors with unique barcodes were added to the restriction fragments for each individual. The final ligates from 12 individuals were pooled, and then the sizes between 220 to 500 bp were separated on 2% agarose gel. Finally, the purified fragments were amplified and the sizes ranged from 270 to 550 bp were purified with a Qiagen gel purification kit and submitted for sequencing. The sequencing was performed on Hiseq2000 platform with paired-end reads of 90 bp.

### SNP discovery and genotyping

The sequencing procedure was performed when the sequence libraries were constructed.. After getting the raw data for each individual, the 5 bp barcode and the 5 bp on the 3′ end of sequences were trimmed and the remaining 80 nucleotides of each PE read were kept for further analysis.

The genome-wide SNP discovery was performed using the RFAP tool pipeline, which included assembly of a pseudo-reference sequence, SNP discovery, genotyping and discrimination of allelic SNPs [29]. Next, the flanking sequences were used to perform BLAST searches against the known reference genome (https://phytozome.jgi.doe.gov/pz/portal.html) [23]. Additionally, previously published SNP sequences and SSR primer sequences from linkage maps [13, 42, 43] were collected for BLAST searches against the reference genome [23]. All of the SNP marker information was listed in Additional file 2.

### Individual linkage map construction and consensus linkage map integration

When the BLAST results for SNPs collected in this study matched known SNP or SSR locus information, the SNPs could be anchored to specific LGs. After calculating the allelic SNPs, all SNP loci with less than 25% maximum missing data and without distorted segregation by Chi-square test with 1: 1 were used for linkage map construction. The MSTMap software [44] was used to construct a high-density genetic map. All the loci were first partitioned into LGs. The major parameters for loci partitioning were as follows: distance_function, Kosambi; p_value, 0.0000001; no_map_dist, 10; missing_threshold, 0.25; objective_function, COUNT. After the individual linkage maps were constructed, the Mergemap software (http://alumni.cs.ucr.edu/~yonghui/mgmap.html) was used to integrate the two individual maps.

### QTL mapping for plant height (PH) and technical length (TL) traits in the two populations

QTL mapping analysis was first done for the two related populations. QTL mapping was performed using the composite interval method (CIM) with the WinQTL cartographer 2.5 software [45]. CIM was used to scan the genetic map and estimate the likelihood of a QTL and its corresponding effect every 2 cM. The forward and backward regression algorithm was used to get cofactors. After initial QTL mapping analysis, a permutation test was done to get the LOD threshold value for each QTL. After QTLs were extracted for each of the population, the QTLs were combined in the consensus linkage map. Then, the flanking sequences of all the SNP markers in the QTL confidence interval were used to blast with the flax genome sequence(https://phytozome.jgi.doe.gov/pz/portal.html), and then the genes located in the confidence interval regions were considered as the potential candidate genes of QTLs.

## Additional files

Additional file 1: The consensus linkage map from the two populations. The linkage map consisted of 15LGs. The markers in the LG with asterisk were the common markers which are used for marker integration. (PDF 2076 kb)

Additional file 2: SNP marker information for consensus linkage map from PH and MH populations (XLSX 712 kb)

Additional file 3: Detailed environmental information of the 4 locations (XLSX 9 kb)

## Abbreviations

CDS: coding sequence
PCR: polymerase chain reaction
PH: plant height
QTL: quantitative trait locus
RIL: recombinant inbred line
SNP: single nucleotide polymorphism
TL: technical length
