# Supplementary figures and images for: Consensus genetic linkage map construction and QTL mapping for plant height-related traits in linseed flax (Linum usitatissimum L.)
Source: BMC Plant Biol. 2018 Aug 7;18:160. doi: 10.1186/s12870-018-1366-6 (PMC6081803; doi:10.1186/s12870-018-1366-6)

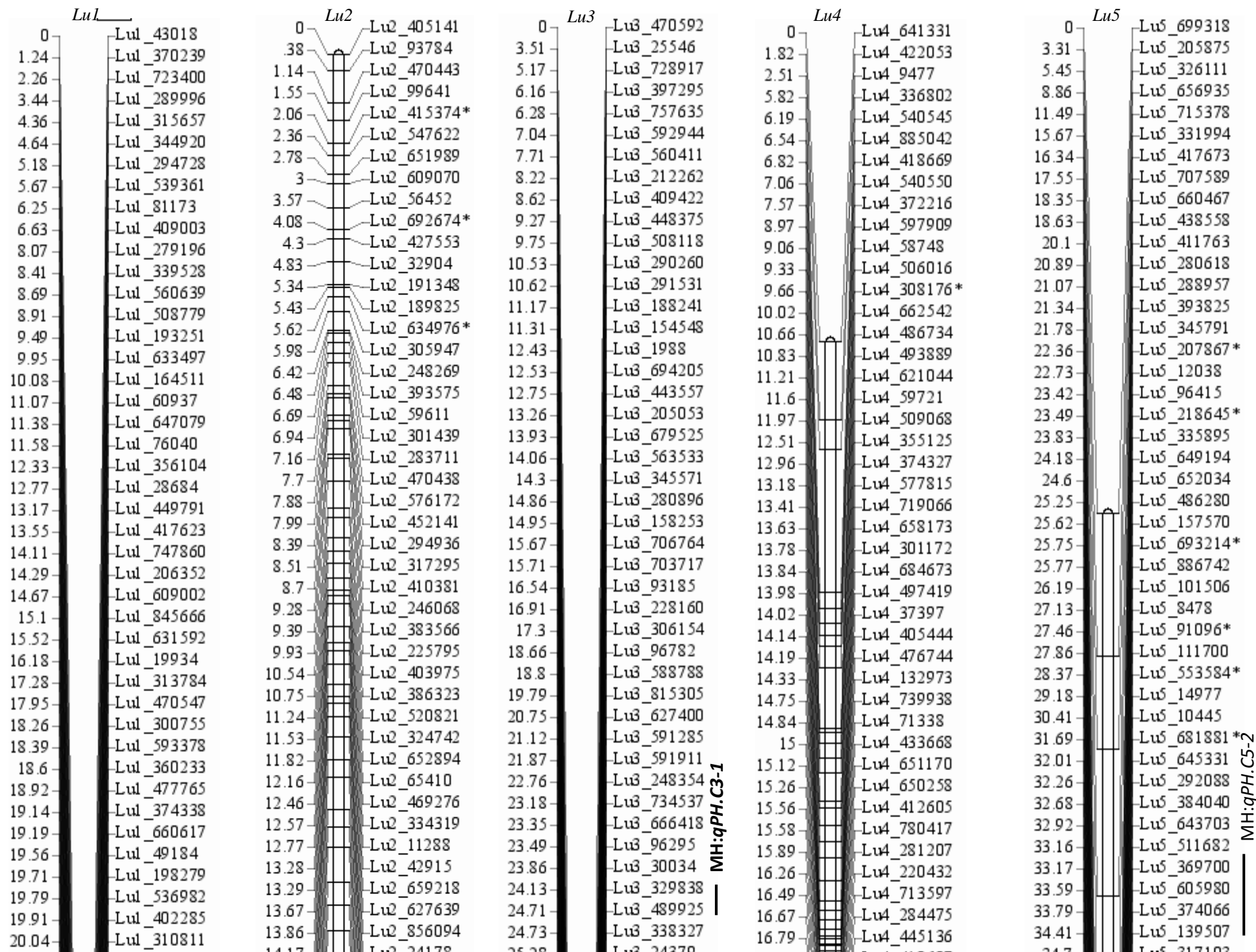

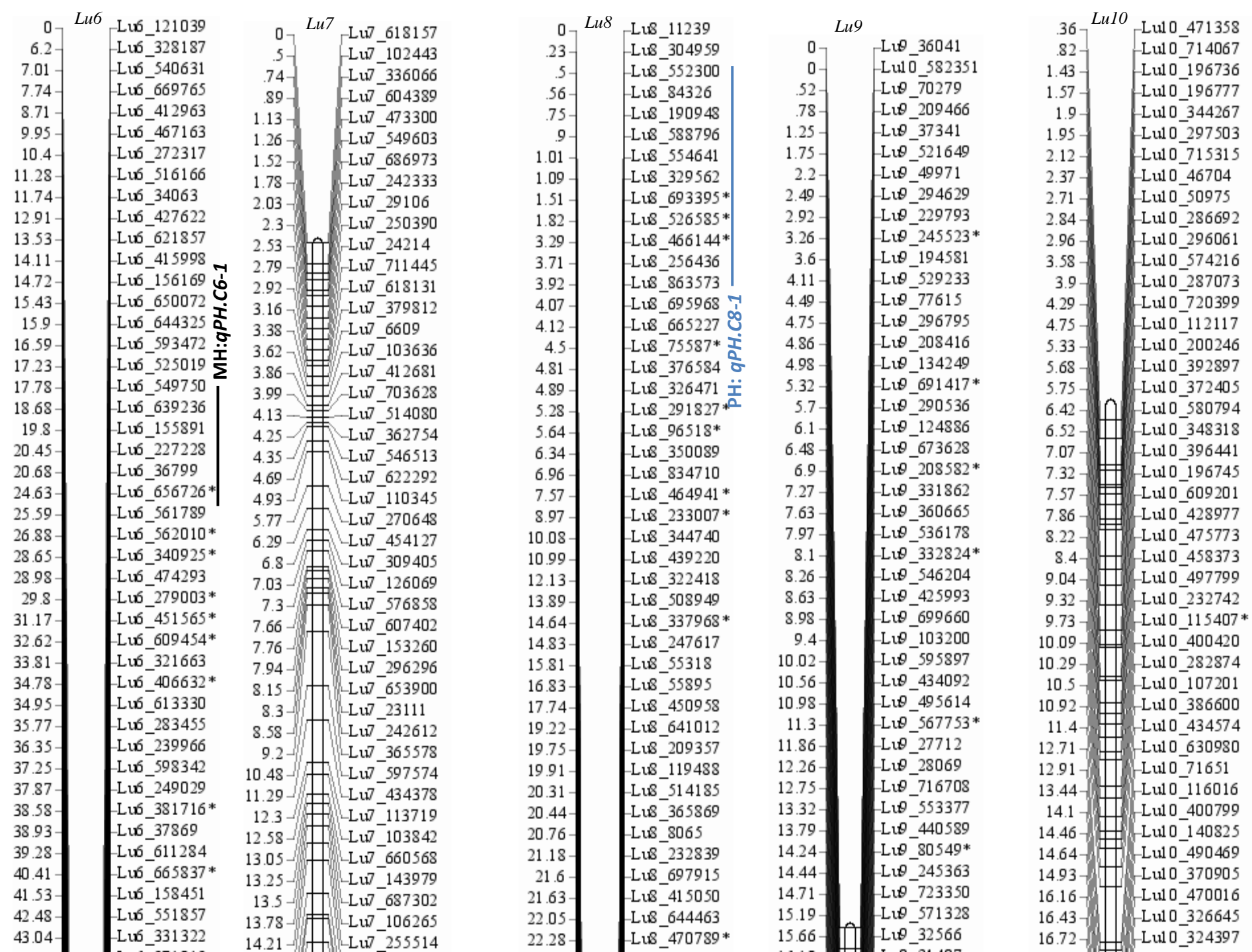

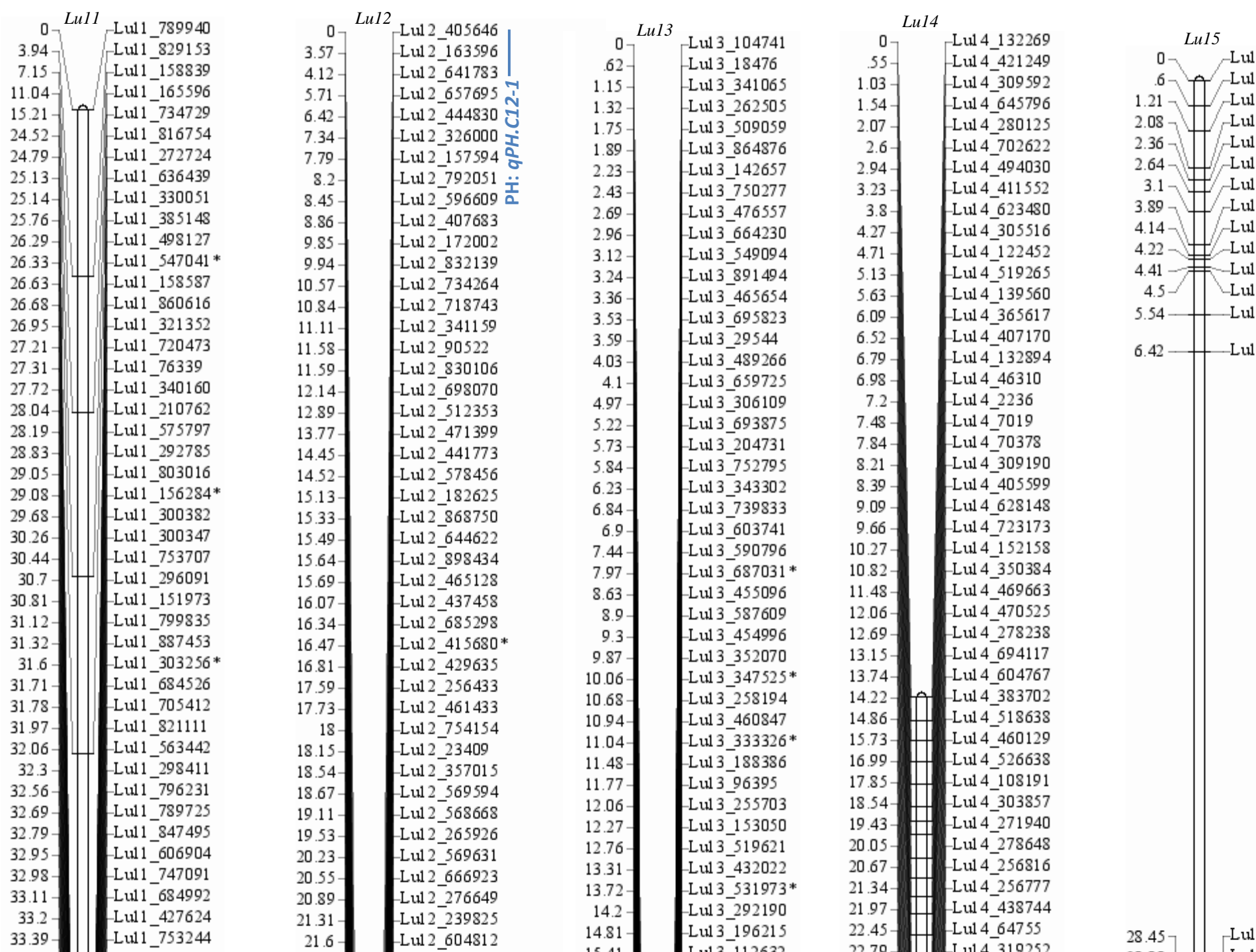

Supplement: Supplementary file 1 — The consensus linkage map from the two populations. The linkage map consisted of 15LGs. The markers in the LG with asterisk were the common markers which are used for marker integration. (PDF 2076 kb) [file 12870_2018_1366_MOESM1_ESM.pdf]
